# The augment of regulatory T cells undermines the efficacy of anti-PD-L1 treatment in cervical cancer

**DOI:** 10.1186/s12865-021-00451-7

**Published:** 2021-09-03

**Authors:** Fengying Xu, Fengying Zhang, Qian Wang, Ying Xu, Shuifang Xu, Caihong Zhang, Lihua Wang

**Affiliations:** 1 Department of Gynaecology and Obstetrics, Jinshan District Tinglin Hospital, Shanghai, 201505 China; 2 Department of Pathology, Jinshan District Tinglin Hospital, Shanghai, 201505 China; 3 Department of Gynecologic Oncology, International Peace Maternity & Child Health Hospital, Shanghai JiaoTong University School of Medicine, 910 Hengshan Road, Xuhui District, Shanghai, 200030 China

## Abstract

**Background:**

Immune checkpoint inhibitors have aroused great expectation of tumor eradication. However, the effect of anti-PD-L1 treatment for cervical cancer is unsatisfactory and the underlying antagonist to anti-PD-L1 efficacy is remained to be studied. Here, we investigated the anti-tumor effect of anti-PD-L1 treatment in cervical tumor model and identified the antagonist to the therapeutic efficacy of anti-PD-L1 treatment.

**Results:**

We found that PD-L1 exhibited a moderate expression in both cervical tumor cell lines and clinical samples compared to other tumor types and the para-tumor tissue respectively. Interestingly, our results showed that the anti-PD-L1 treated mice were dichotomously divided into responsive and unresponsive group after five cycles of anti-PD-L1 treatment although all the mice had the same genome background. In addition, the unresponsive tumors showed less tumor necrosis area and higher immunosuppression activity induced by regulatory T cells (Tregs) population than the responsive ones. Furthermore, we found that anti-PD-L1 treatment autonomously upregulated Tregs proliferation and frequency in multiple immune organs, and, most importantly, Tregs depletion significantly depressed the tumor growth rate and tumor weight compared with either anti-PD-L1 or anti-CD25 treatment alone. Finally, we observed that the upregulating effector CD8^+^ T cell is associated with the better therapeutic effect of anti-PD-L1 therapy post Tregs depletion.

**Conclusions:**

Anti-PD-L1 treatment upregulates Tregs frequency and proliferation in tumor model, and the depletion of Tregs may be a useful adjuvant strategy for anti-PD-L1 therapy of cervical cancer.

**Supplementary Information:**

The online version contains supplementary material available at 10.1186/s12865-021-00451-7.

## Introduction

Cervical cancer, as the second most malignant gynecological tumor with high incidence and high mortality among women, severely threats women’s health all around the world [1, 2]. One of the most important reasons for the increasing trend of cervical cancer is the higher frequency exposure to human papillomavirus (HPV) caused by the bad sexual habits, such as early beginning of sexual activities and multiple partners [3]. Currently, the main therapy for cervical cancer includes radiotherapy, chemotherapy, surgery and targeted therapy [4]. However, both radiotherapy and chemotherapy caused serious side-effects, such as hair loss, nausea, anorexia and diarrhea, which undermines the quality of patient life. Besides, the common total radical hysterectomy and bilateral pelvic lymphadenectomy surgery may by futile for patients with metastasis or at advance stage [5]. Although EGFR and COX-2 mediated targeted therapy have been used for the treatment of cervical cancer, the survival rate and prognosis of cervical patients were not significantly improved [6, 7].

The initiation and progression of cervical cancer are associated with the immunosuppression on CD4^+^ and CD8^+^ T cells caused by HPV infection. Recently, immune checkpoint inhibitors, such as PD-1, PD-L1 and CTLA-4, have been intensely studied in many solid tumors, and many clinical trials have shown the long-lasting improved prognosis of patients, especially in melanoma and lung cancer [8–10]. As for cervical cancer, anti-CTLA-4 showed little clinical efficacy in patients with recurrence or metastasis [11]. Previous studies showed that tumoral PD-L1 expression was observed in 72% cervical and vulvar squamous carcinomas (SCC) and 95% cervical intraepithelial neoplasias (CINs) [9, 12]. Meanwhile, Meng et al. reported that 61% (59/97) of the patients exhibited PD-1 expression in the tumor stroma of cervical cancer [13]. Pembrolizumab (anti-PD-1) also had been approved by FDA for advanced cervical cancer, and the clinical studies have demonstrated that pembrolizumab demonstrated antitumor activity and exhibited a safety profile in patients with programmed death ligand 1-positive advanced cervical cancer [14, 15]. Even so, the overall response rate was only 14.3–17%, and 75% patients experienced treatment related adverse events, such as rash and pyrexia [14, 16]. Therefore, the therapeutic efficiency of anti-PD-L1 treatment is urgent to be improved in advanced cervical cancer.

Regulatory T cells (Tregs), defined by CD3^+^CD4^+^CD25^−^FOXP3^+^, played an important role in immune escape and thus undermined the therapeutic efficacy of immunotherapy in various tumor types. Previous studies showed that TNFR2^+^ Tregs increased in tumors of cervical cancer patients, and Foxp3^+^ tumor infiltrating immune cells in the central tumor area might be a biomarker for risk stratification in cervical cancer patients [17–19]. In contrast, Simone Punt et al. reported that a high total number of Tregs were significantly correlated with improved disease-specific and disease-free survival in cervical adenocarcinoma [20]. However, the effect of anti-PD-L1 on Tregs levels and functions is not clear in cervical cancer. Here, we explore the effect of PD-L1 treatment in syngeneic cervical tumor model and investigated the potential role of Tregs in undermining the effect of anti-PD-L1 therapy in cervical cancer.

## Methods

### Cells and regents

U14 and Hela cell lines were purchased from ATCC, and were cultured in DMEM supplemented with 10% FBS + 1% penicillin/streptomycin antibiotics.

### Patient samples and mouse model

Six clinical samples of cervical cancer patients were collected from the department of gynaecology and obstetrics, Jinshan district, Tinglin hospital. Written informed consent forms were obtained from all the patients. The study was approved by the Ethics Committee of the Institute of the department of gynaecology and obstetrics, Jinshan district, Tinglin hospital.

The 6–8 weeks age C57BL/6J mouse used for tumor model was bought from Nanjing Model Animal Center in China. All procedures about mice were approved by the Animal Ethics Committee of shanghai Jiaotong University.

### Protein extraction and western blot

For U14 and Hela cell lines, the collected cells were washed by cold PBS for two times. 2 × 10^6^ cells were treated by RAPA buffer on ice for 30 min. Then, the lysate was centrifuged for 15 min at 12,000*g* at 4 °C. Then, the protein concentration was measured by BCA method. The loading sample was made at the concentration of 1 μg/μl and were loaded for 10 μl in SDS-PAGE gel and conducted protein transfer with NC membrane. The targets bands were cut and blocked by 5% skim milk for 1 h at room temperature. Then, the bands were washed by TBST for 3 times, and were incubated by PD-L1 antibody(ab213524, 1:1000) overnight at 4 °C. The bands were washed by TBST for 3 times, and were incubated by the second antibody for 1 h at room temperature. Finally, the blotting signal was recorded under machine.

### In vivo tumor progression and immunotherapy models

All the immune competent C57BL/6J SPF mice were purchased from Nanjing model animal center and feed in the facility of Shanghai Jiaotong University. numbered and randomly assigned into different groups. 3 × 10^6^ logarithmic growth phase U14 cells were transplanted subcutaneously into the flanks of 7-week-old C57BL/6 female mice. For the mouse model in Fig. 2, both PBS and anti PD-L1 treatment groups include six mice; for the mouse model in Fig. 4, sixteen mice were equally assigned to PBS, anti-PD-L1, anti-CD25 and anti-PD-L1 plus anti-CD25 groups, and all these group were treated with corresponding regents every two days. The tumor size was measured seven days post tumor challenge with a caliper every 2–3 days, and tumor volume was calculated by width^2^ × length × 0.5. Mice were sacrificed according to the animal welfare requirement at the endpoint (The maximum tumor less than 15 mm in diameter). The death of mouse during treatment was used as the exclusion criteria.

### RNA extraction and RT-PCR

The cells were harvested and washed for two time with cold PBS. 1 ml Trizol reagent was added in 2 × 10^6^ cells and sufficiently suspended. The total RNA was extract according established protocol. In quantitative PCR (q-PCR), the reverse transcription of 1.5 μg total RNA were conducted by using SuperScript III First-Strand Synthesis System. The harvested cDNA was diluted for five times by ddH_2_O. The SYBR Green PCR Master Mix (Applied Biosystems) was used for qPCR, and three repeats were assigned in a Real-Time PCR System (Applied Biosystems). All used primers for qPCR are listed as follow: mouse-PD-L1: forward-5′- GCTCCAAAGGACTTGTACGTG-3′; reverse-3′-TGATCTGAAGGGCAGCATTTC-5′; human-PD-L1: forward-5′- GCTGCACTAATTGTCTATTGGGA-3′; reverse-3′-AATTCGCTTGTAGTCGGCACC-5′; mouse-GAPDH: forward-5′-GAAGGTCGGTGTGAACGGAT-3′; reverse-3′-TGATGGGCTTCCCGTTGATG-5′; human-GAPDH: forward-5′-CGGATTTGGTCGTATTGGG-3′; reverse-3′-CTCGCTCCTGGAAGATGG-5′; mouse-Ki67: forward-5′-ATCATTGACCGCTCCTTTAGGT-3′; reverse-3′-GCTCGCCTTGATGGTTCCT-5′. mouse-CD206: forward-5′-CTCTGTTCAGCTATTGGACGC-3′; reverse-3′-CGGAATTTCTGGGATTCAGCTTC-5′; mouse-Ly6G: forward-5′-GACTTCCTGCAACACAACTACC-3′; reverse-3′-ACAGCATTACCAGTGATCTCAGT-5′; mouse-Arginase1: forward-5′-TGTCCCTAATGACAGCTCCTT-3; reverse-3′-GGAGCTGTCATTAGGGACATCA-5′; mouse-FOXP3: forward-5′-CCCATCCCCAGGAGTCTTG-3′; reverse-3′-ACCATGACTAGGGGCACTGTA-5′; mouse-Ly6c: forward-5′-GCAGTGCTACGAGTGCTATGG-3′, reverse-3′-ACTGACGGGTCTTTAGTTTCCTT-5′.

### Immunohistochemistry

Tumor and spleen tissue samples were carefully resected and immediately fixed in 4% paraformaldehyde overnight at room temperature. The fixed tissues were embedded in standard paraffin wax to product 5-μm sections for HE and immunohistochemistry assay. In brief, the tissue sections were deparaffinized in xylene for 3 times (10 min/time) and rehydrated via an ethanol gradient (100%, 95%, 80%, 75%, 50%). After antigen retrieval with pH 6.0 citrate buffer, sections were incubated in a 0.3% H_2_O_2_ solution to remove peroxidase at room temperature for 10 min. Then, the sections were washed by PBS for 3times (10 min/time) and blocked by normal goat serum or 5% BSA for 1 h at 37 °C. The sections were then incubated with rat anti-mouse Foxp3 monoclonal antibody (ab215206, 1:200) or anti- PD-L1 (ab213524, 1:200) at 4 °C overnight. On the second day, the tissue sections were treated with instant SABC kit according to provided protocol. Finally, the sections were stained with hematoxylin and sealed for observation under microscope.

### In vivo antibody treatment

3 × 10^6^ U14 cells were subcutaneously injected as described above. Seven days post tumor cell injection, anti-PD-L1 antibody [In vivo mab anti-mouse PD-L1 (B7-H1), BioX Cell, BE0101] and anti-CD25 (IL-2Rα, In vivo plus anti-mouse CD25, BioX Cell, BP0012) were intraperitoneally injected (200 μg per dose per mouse) as indicated schedule. Mice were euthanized and tumors were harvested after five times’ antibody injections. The resected tumors were photographed and measured.

### Cell and tissue FACS analysis

The peripheral blood, tumor, draining lymph node and spleen were isolated from mice. Then, the single cell suspension for these sample were prepared. The single cells suspension was stained with the following antibodies: anti-mouse CD25-BV605, CD3-FITC and CD4-pacific blue were stained for 30 min at 4 °C, and the samples were treated with fixation/permeabilization solution for 40 min in dark. Then, the fixed cells were stained by FOXP3-PE and anti-Ki67 for 30 min at 4 °C. Finally, the samples were washed for two times by cold PBS. For the sorting of Tregs, the staining panel is the same as mentioned above. The isolated cells were resuspended with 1 ml Trizol regent and the total RNA was extract for RT-PCR.

### Statistical analysis

All experiments were repeated three times for statistical analyses. Mice were randomly allocated to experimental and control groups before treatment. Normally distributed data were analyzed by unpaired two-tailed Student’s t-test for single comparisons. Two-way ANOVA test was used between groups. A P-value of < 0.05 was considered statistically significant.

## Results

### PD-L1 exhibited high expression in cervical tumor cell lines and tumor tissue

**Fig. 1:**
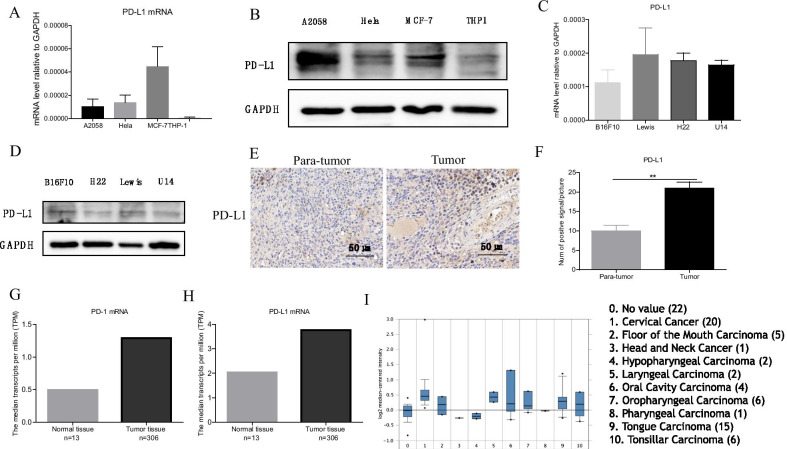
PD-L1 exhibited high expression in tumor cell lines and tumor tissue. **A** The mRNA level of PD-L1 in Hela cells and other three human tumor cell lines. **B** The protein level of PD-L1 in Hela cells and other three human tumor cell lines. The uncropped blots provided in Additional file 2: Fig. S1A. **C** The mRNA level of PD-L1 in U14 cells and other three mouse tumor cell lines. **D** The protein level of PD-L1 in U14 cells and other three mouse tumor cell lines. The uncropped blots provided in Additional file 2: Fig. S1B. **E** The detection of expression of PD-L1 by IHC in human cervical tumor tissue. The pictures were magnified for 400 times. Scale bar: 50 μm. **F** The counting of positive cells in figure (**E**). **G**, **H**. The mRNA level of PD-1 (**F**) and PD-L1 (**G**) in cervical squamous cell carcinoma patients. The original data of the two graphs was analysed by the GEPIA database for cervical squamous cell carcinoma. **I** PD-L1 expression in different types of cancer. The original information of this graph comes from the Oncomine database

The expression of PD-1 and PD-L1 are the important predicative biomarker for anti-PD-1/L1 therapeutic efficacy. Therefore, we first investigated the expression of PD-L1 expression in human and mouse cervical cell lines. Our results showed that Hela and U14 cell lines had moderate PD-L1 expression in mRNA (Fig. 1A, C) and protein level (Fig. 1B, D, Additional file 1) compared with other tumor types, indicating the potential therapeutic effect of anti-PD-L1 therapy in cervical cancer. To investigate the expression of PD-1 and PD-L1 expression in cervical patient samples, we conducted immunohistochemistry for PD-1/L1 in the tumor tissue and corresponding para-tumor tissue of cervical patients. We found that the expression PD-L1 significantly increase in tumor tissue compared to the corresponding para-tumor tissue of patients (Fig. 1E, F). In addition, we analyzed the mRNA level of PD-1 and PD-L1 in human cervical squamous cell carcinoma by GEPIA database and Oncomine database. We observed that PD-1 (Fig. 1G) and PD-L1 (Fig. 1H) showed higher expression in cancer patients than the health controls, and cervical cancer had a relative high PD-L1 expression compared with other common cancer types (Fig. 1I). Taken together, the PD-L1 therapy may be a promising option for the treatment of cervical tumor.

### The anti-tumor effect of anti-PD-L1 therapy was undermined by the enhanced immunosuppression in tumor

**Fig. 2:**
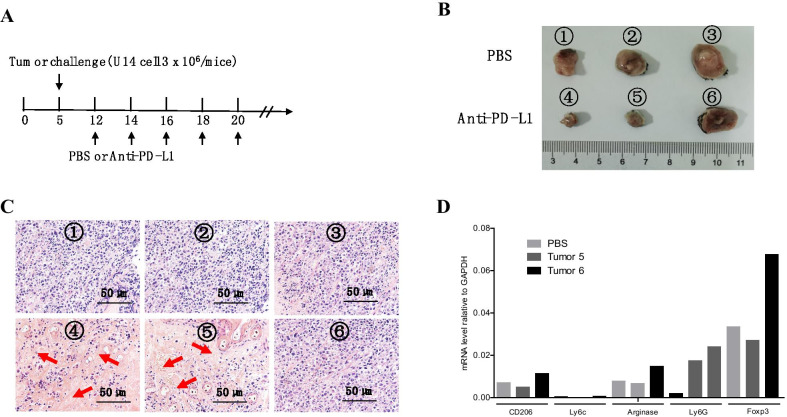
The anti-tumor effect of anti-PD-L1 treatment was undermined by the enhanced immunosuppression in tumor. **A** The anti-PD-L1 treatment schedule in syngeneic tumor mouse model. 3 × 10^6^ U14 cervical cells was subcutaneously injected on the flank of 7-weeks C57BL/6 mouse. Anti-PD-L1 (200 μg/time/mouse) was intraperitoneally injected in mouse seven days post tumor challenge, and five injection were performed. **B** The representative picture of tumors after five times anti-PD-L1 therapy. The tumor was numbered with Arabic numerals. **C** The HE dying for the tumors in figure (**B**), and the red arrow represents the necrosis area. The pictures were magnified for 400 times. Scale bar: 50 μm. **D** The mRNA level of immunosuppressive molecular, including CD206, Ly6C, Ly6G, Foxp3, and Arginase in control (no PD-L1 treatment), tumor ⑤ and tumor ⑥

To investigate the effect of anti-PD-L1 on tumorigenesis of cervical cancer, we constructed the syngeneic tumor model in immune competent C57BL/6 mouse. Anti-PD-L1 or PBS was administrated according the treatment schedule (Fig. 2A). We found that U14 cell line had 100% tumor formation rate in C57BL/6 mice. Anti-PD-L1 treatment significantly depressed the growth of xenografted tumor in most of mice (Fig. 2B). In contrast, 30% mice were not response to anti-PD-L1 treatment. Then, we conducted HE dye to further investigated the tumor microenvironment situation in responding and non-responding tumors. We found that the tumors responding to anti-PD-L1 treatment showed the higher levels of tumor necrosis than that of unresponsive ones (Fig. 2C). Numerous studies have shown that the immunosuppression activity in tumor microenvironment severely undermined the therapeutic effect of PD-L1 treatment in many types of tumor. Therefore, we extracted mRNA from responsive and unresponsive tumors and detected the immunosuppressive activity by several vital molecules, including Foxp3, CD206, Arginase, Ly6c and Ly6G. Our results showed that the unresponsive tumors showed higher immunosuppressive activity than the responsive one (Fig. 2D). Collectively, the excessive upregulation of Tregs level after anti-PD-L1 treatment may undermine the therapeutic efficiency.

### The upregulating Tregs in tumors was associated with the compromised therapeutic efficiency of PD-L1 treatment

**Fig. 3:**
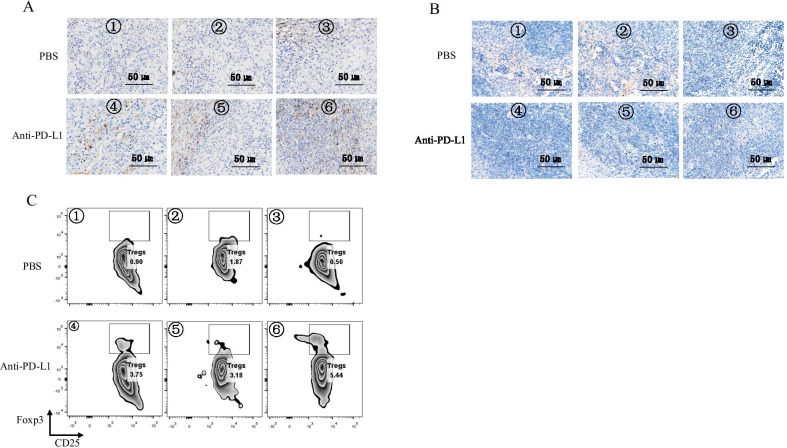
The upregulating Tregs in tumors was associated with the compromised therapeutic efficiency of PD-L1 treatment. **A** The Foxp3 IHC assay for the tumor tissue in (**B**). The pictures were magnified for 400 times. Scale bar: 50 μm. **B** The Foxp3 IHC assay for the spleen in the corresponding host of tumors in (**B**). The pictures were magnified for 400 times. Scale bar: 50 μm. **C** The detection of Tregs in the tumors of (**B**) with flow cytometry. The Tregs was defined as CD45^+^CD3^+^CD4^+^CD25^+^Foxp3^+^

As shown in the Fig. 2D, the unresponsive tumor showed very high level of Foxp3 compared to the responsive tumor and other immunosuppressive markers. Therefore, we hypothesized that the upregulated Tregs level might account for the compromised anti-tumor effect in unresponsive ones. The IHC results showed that Tregs had a relatively high level in unresponsive tumors (Fig. 3A) and corresponding spleens (Fig. 3B) compared to responsive tumors. To further identified our finding, we conducted the flow cytometry to detect the frequency of Tregs in tumors. Consistently, we indeed observed the highest Tregs frequency in unresponsive tumor (Fig. 3C). Of note, we also found that anti-PD-L1 promoted the frequency of Tregs in both responsive and unresponsive tumors at different degree (Fig. 3C).

### Tregs depletion strengthened the anti-tumor effect of anti-PD-L1 treatment in cervical tumor model

**Fig. 4:**
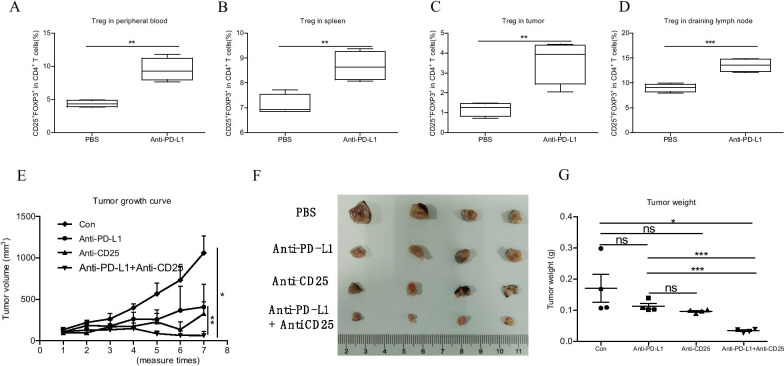
Tregs depletion strengthened the anti-tumor effect of anti-PD-L1 treatment in cervical tumor model. **A**–**D** The change of Tregs level in peripheral blood (**A**), spleen (**B**), tumor (**C**) and DLN (**D**) in the syngeneic tumor model. The above samples were collected at the endpoint. N = 4. Two-tailed unpaired T-test was performed. **E** The tumor growth curve under different treatment. The tumor was measured with a caliper, and the tumor volumes were calculated with the formula: ½ *length *width*width. Two-tailed unpaired T-test was performed. **F** The tumor pictures after different treatment. N = 4. **G** The tumor weight in different groups. n = 4. The tumors were weight with a analytical balance. Two-tailed unpaired T-test was performed. ns: no significant difference, *p < 0.05, **p < 0.01compared to the control groups. A *p* value less than 0.05 was considered to be statistically significant

Although anti-PD-L1 could effectively depressed the growth rate of tumor in the mouse model, only 20% reduction of tumor weight was achieved. Therefore, we hypothesized that Tregs depletion could enhance anti-PD-L1 efficacy. Therefore, we used PBS, anti-PD-L1, anti-CD25 or anti-PD-L1 plus anti-CD25 to treated cervical tumor mouse model (Additional file 2: Fig. S1A). Then, we performed C-flow cytometry of Tregs population in the various immune organs after several five times immunotherapy (Additional file 2: Fig. S1B). Our results showed that anti-PD-L1 significantly increased the percentage of Tregs in peripheral blood (Fig. 4A), spleen (Fig. 4B), tumors (Fig. 4C) and lymph node (Fig. 4D). Importantly, anti-PD-L1 plus anti-CD25 treatment significantly inhibited the growth of syngeneic tumor compared to PBS or anti-PD-L1 or anti-CD25 alone (Fig. 4E). The tumors were harvest at the endpoint, and the tumor weight in anti-PD-L1 plus anti-CD25 treatment group was significantly smaller than the control group or anti-PD-L1 or anti-CD25 group alone (Fig. 4F, G). Taken together, Tregs depletion could strengthen the therapeutic effect of anti-PD-L1 treatment by decreasing upregulating immunosuppression after immunotherapy.

### The increased Tregs proliferation depressed the level of effector CD8^+^ T cells after PD-L1 treatment

**Fig. 5:**
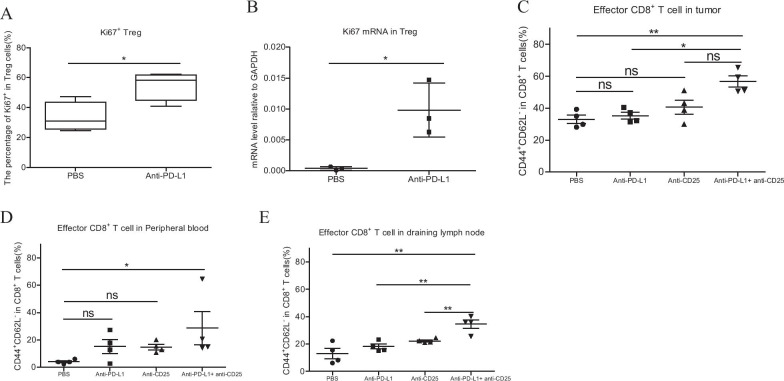
The increased Tregs proliferation depressed the level of effector CD8^+^ T cells after PD-L1 treatment. **A** The change of Ki67^+^ Tregs in the peripheral blood of mice after anti-PD-L1 treatment. The blood was collected at the endpoint. N = 4. Two-tailed unpaired T-test was performed. **B** The mRNA level of Ki67 in sorted Tregs cells from the spleen of mouse. N = 3. Two-tailed unpaired T-test was performed. **C**–**E** The change of the frequency of effector CD8^+^ T cells in tumor (**C**), the peripheral blood (**D**) and DLN (**E**) at the endpoint. N = 4. The effector CD8 + T cell was defined as CD3^+^CD8^+^CD62L^−^CD44^+^. Two-tailed unpaired T-test was performed. ns: no significant difference, *p < 0.05, **p < 0.01compared to the control groups. A *p* value less than 0.05 was considered to be statistically significant

To figure out the reason for the increase of Tregs, we analyzed the signature of Tregs after anti-PD-L1 treatment. We found that anti-PD-L1 treatment significantly upregulated the percentage of Ki67^+^ Tregs, indicating the increasing Tregs proliferation (Fig. 5A). Additionally, we also sorted Tregs for PBS and PD-L1 treated group to detect the mRNA level of Ki67 transcription. Consistently, Ki67 showed the higher mRNA level in anti-PD-L1 group compared with the PBS group (Fig. 5B). Therefore, the increased Tregs after anti-PD-L1 therapy may associated with increasing proliferation of Tregs.

Considering of the important role of effector CD8^+^ T cells (defined by CD3^+^CD8^+^CD62L^−^CD44^+^) in anti-tumor response. We respectively analyzed the frequency of effector T cells after PBS, or anti-PD-L1 or anti-CD25 or anti-PD-L1 plus anti-CD25 treatment. Our results that anti-PD-L1 plus anti-CD25 treatment group had a significantly higher level of effector CD8^+^ T cells than PBS and PD-L1 group (Fig. 5C, D). In the draining lymph node, we also observed the more distinct increase of effector CD8^+^ T cells in the combination group compared to any of the other three groups (Fig. 5E). In conclusion, the increased effector CD8^+^ T cells may be associated with the better therapeutic effect after Tregs depletion in cervical tumor model.

## Discussion

Currently, immunotherapy had aroused the widely concern of researchers focusing on various tumor types [21]. Although several clinical trials had verified the effect of anti-PD-L1 on advanced tumors, most of patients had great difficulty in maintaining the long-lasting response to immune checkpoint mediated immunotherapy, let alone the eradicating of tumor cells [8, 11]. However, the underlying mechanism for the low anti-tumor efficacy of immunotherapy in cervical cancer was not very clear. Here, we found that anti-PD-L1 dichotomously affected tumor growth in the syngeneic mouse model, and the different response to anti-PD-L1 treatment is associated with the autonomously increased Tregs proliferation and frequency in multiple immune organs and tumors. We also found that Tregs depletion significantly enhanced the tumor depression effect of anti-PD-L1 treatment in vivo. Therefore, our research provided a novel insight for the limited anti-tumor efficacy of anti-PD-L1 treatment in cervical cancer.

The increased Tregs may be one of the important mechanisms of cervical tumors to resist immunotherapy efficacy. Although we failed to explore the underlying mechanism for Tregs increasing post PD-L1 treatment, we indeed observed the correlation between IL6 expression and Tregs upregulation. Under tumor conditions, various cytokines, such as GM-CSF, IL6, TNF-α, and other chemokines [22, 23]. Previous studies showed that IL6 and Tumor necrosis factor α (TNFα) could promote Tregs proliferation in tumor sites. The recent study reported that anti-PD-L1 treatment or the Rhein plus PD-L1 therapy groups upregulated the IL6 level in the established 4T1 breast cancer xenografts [24]. Consistently, we also observed the slightly increase of IL6 in the tumor tissue after anti-PD-L1 therapy (data not show). TNF is a potent pro-inflammatory cytokine, which played a vital role in the balance of tumor microenvironment. Benoît L Salomon et al. reported that TNF is able to increase expansion, stability, and possibly function of Tregs via TNFR2 [23]. In addition, Lack of interleukin-6 in the tumor microenvironment augments type-1 immunity and increases the efficacy of anti-PD-L1 therapy in CT26 cells mouse model [25]. Collectively, we supposed that the increased Tregs probably caused by increased IL6 expression after PD-L1 treatment, which, however, remained to be further confirmed in the future considering of the limited number of mice response to anti-PD-L1 treatment in our project.

Although we observed the enhanced anti-tumor effect after Tregs depletion during anti-PD-L1 treatment in mouse model, we also should be careful for the quickly use of this strategy in clinical cervical cancer patients. A few studies reported that Tregs depleted mice suffered serious autoimmune disease [26]. Furthermore, anti-PD-L1 also may lead to huge immune storm in the host. Therefore, much more attention should be paid on the treatment related adverse event in cervical cancer patients during Tregs depletion combined anti-PD-L1 treatment in cervical cancer patients in the future. Admittedly, the animal number in the was a little bit small in this project and more animals might be added in every group to make our conclusion even solid in the future.

In conclusion, we found that anti-PD-L1 treatment upregulated Tregs levels in cervical cancer mouse model, and Tregs depletion maybe a promising adjuvant treatment of anti-PD-L1therapy for cervical cancer treatment.

## Supplementary Information

**Additional file 1**. The original, uncropped gels for western blot.

**Additional file 2**. Treatment schedule and the representative figure for Cytometry flow analysis.

## Data Availability

All data and materials were available from the corresponding author.
