# Supplementary material for: The augment of regulatory T cells undermines the efficacy of anti-PD-L1 treatment in cervical cancer
Source: BMC Immunol. 2021 Sep 3;22:60. doi: 10.1186/s12865-021-00451-7 (PMC8414724; doi:10.1186/s12865-021-00451-7)
Supplement: Supplementary file 1 — Additional file 1. The original, uncropped gels for western blot. [file 12865_2021_451_MOESM1_ESM.pptx]

## Slide 1
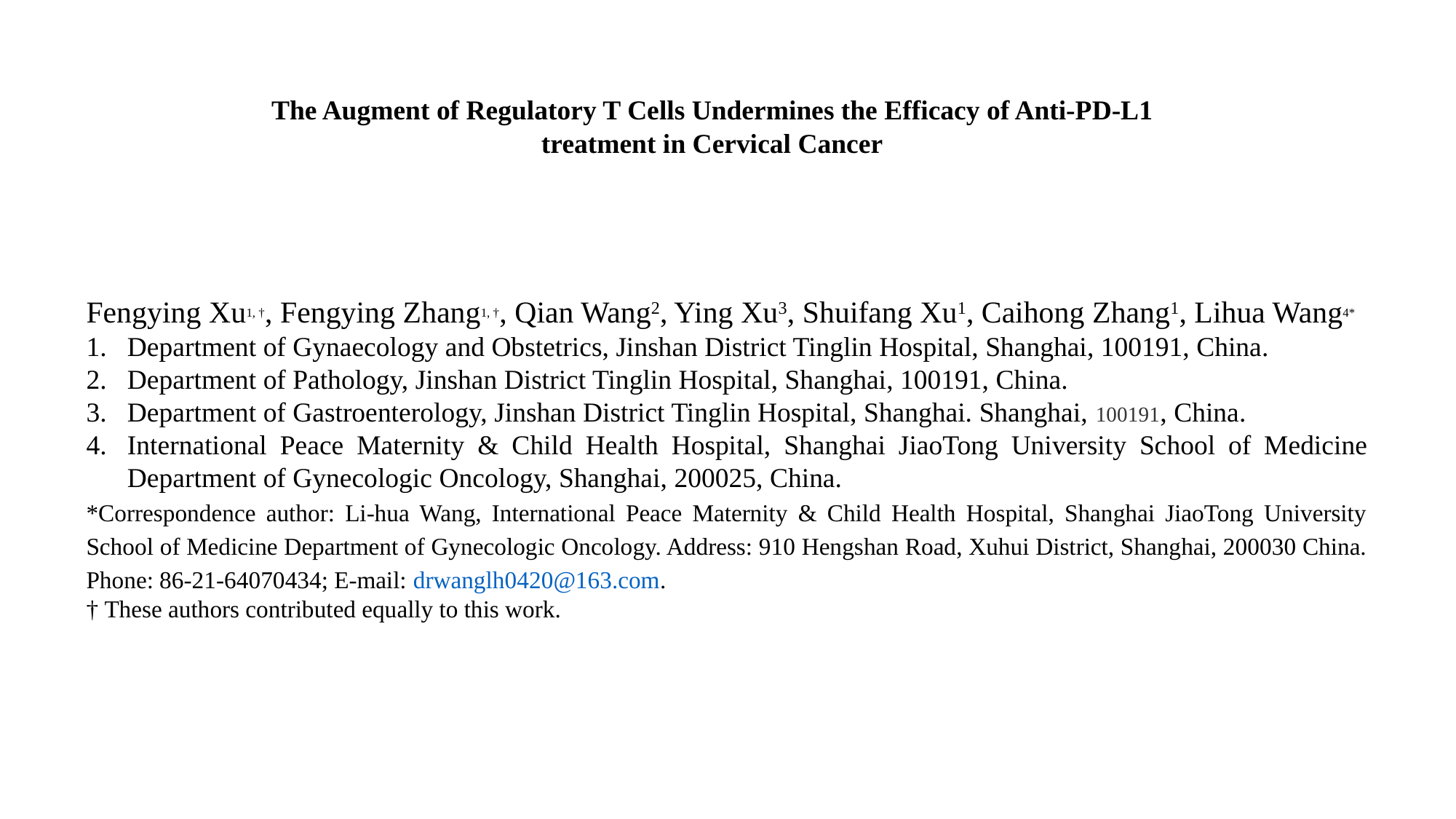

The Augment of Regulatory T Cells Undermines the Efficacy of Anti-PD-L1 treatment in Cervical Cancer
Fengying Xu1, †, Fengying Zhang1, †, Qian Wang2, Ying Xu3, Shuifang Xu1, Caihong Zhang1, Lihua Wang4*
Department of Gynaecology and Obstetrics, Jinshan District Tinglin Hospital, Shanghai, 100191, China.
Department of Pathology, Jinshan District Tinglin Hospital, Shanghai, 100191, China.
Department of Gastroenterology, Jinshan District Tinglin Hospital, Shanghai. Shanghai, 100191, China.
International Peace Maternity & Child Health Hospital, Shanghai JiaoTong University School of Medicine Department of Gynecologic Oncology, Shanghai, 200025, China.
*Correspondence author: Li-hua Wang, International Peace Maternity & Child Health Hospital, Shanghai JiaoTong University School of Medicine Department of Gynecologic Oncology. Address: 910 Hengshan Road, Xuhui District, Shanghai, 200030 China. Phone: 86-21-64070434; E-mail: drwanglh0420@163.com.
† These authors contributed equally to this work.

## Slide 2
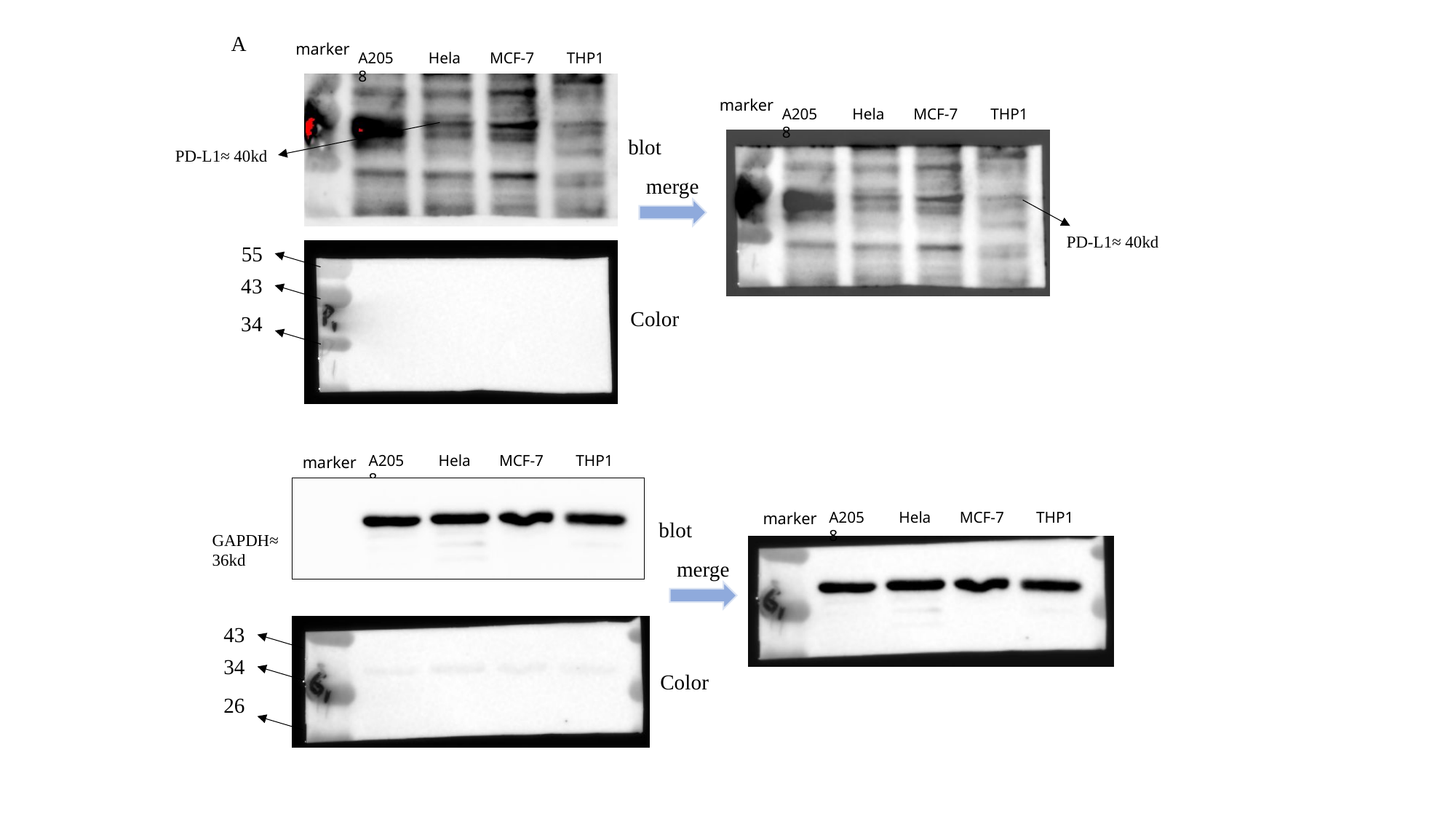

A
marker
A2058
Hela
MCF-7
THP1
marker
A2058
Hela
MCF-7
THP1
blot
PD-L1≈ 40kd
merge
PD-L1≈ 40kd
55
43
Color
34
A2058
Hela
MCF-7
THP1
marker
A2058
Hela
MCF-7
THP1
marker
blot
GAPDH≈ 36kd
merge
43
34
Color
26

## Slide 3
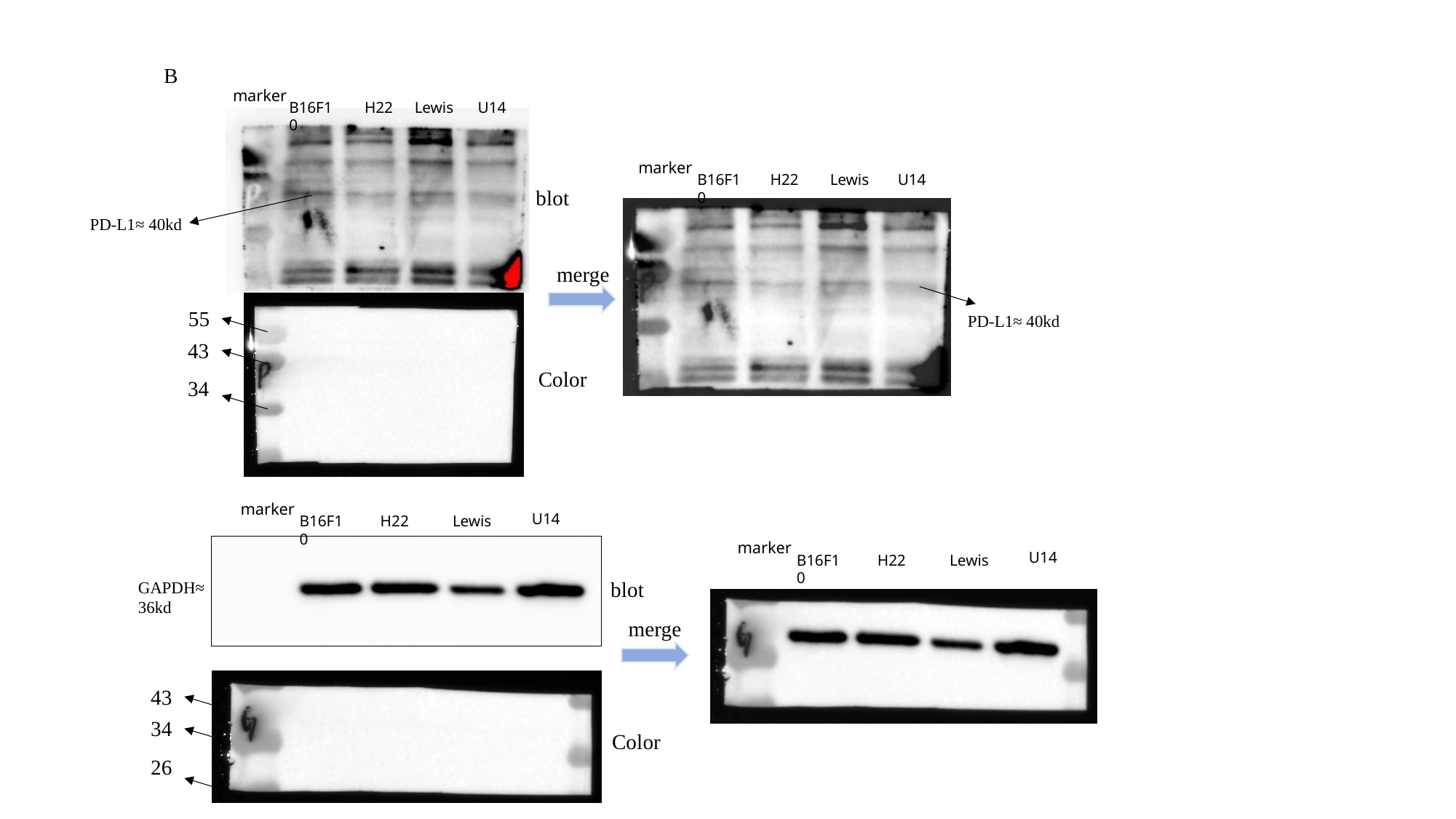

B
marker
B16F10
H22
Lewis
U14
marker
B16F10
H22
Lewis
U14
blot
PD-L1≈ 40kd
merge
55
PD-L1≈ 40kd
43
Color
34
marker
U14
B16F10
H22
Lewis
marker
U14
B16F10
H22
Lewis
blot
GAPDH≈ 36kd
merge
43
34
Color
26

## Slide 4
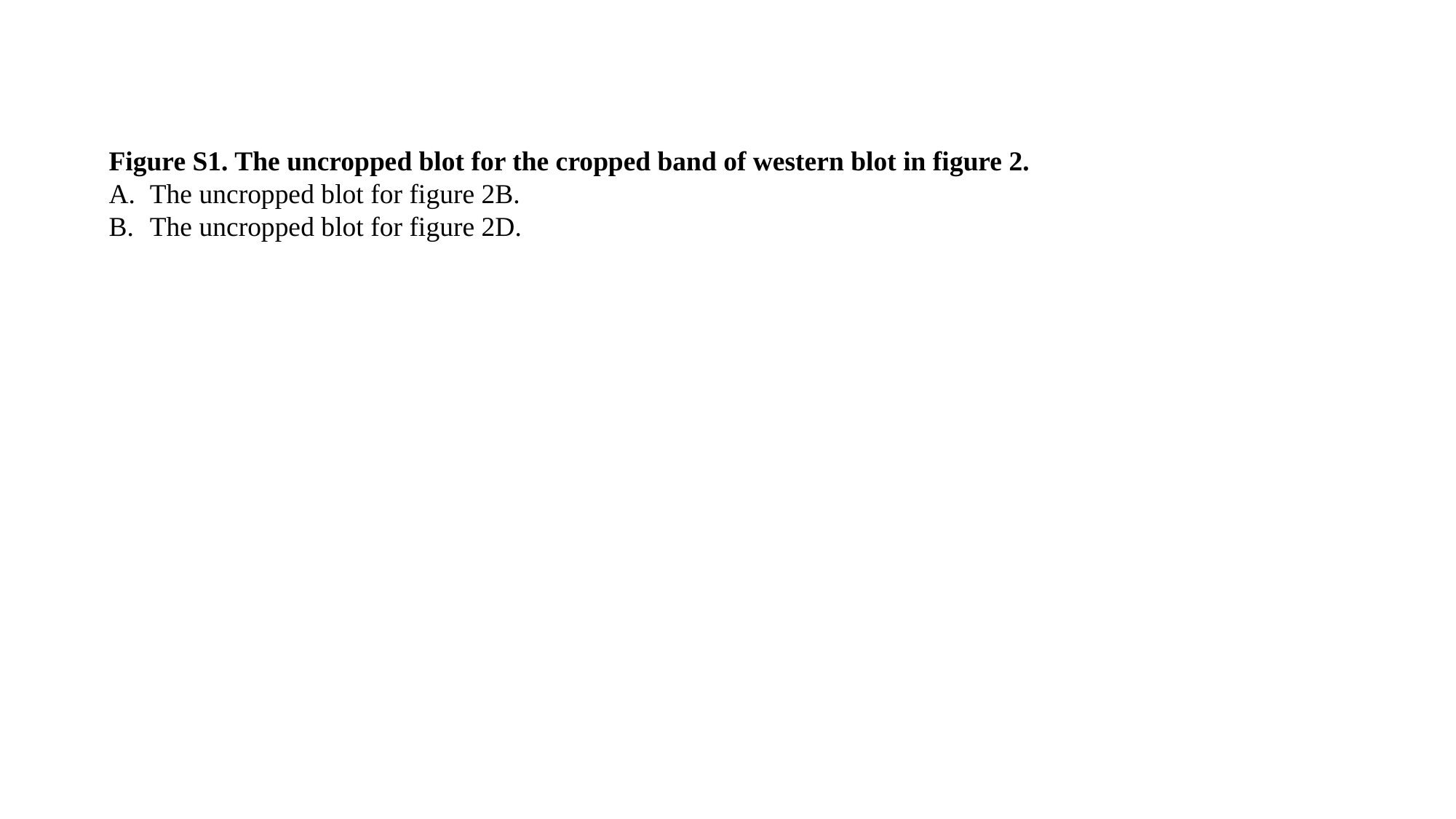

Figure S1. The uncropped blot for the cropped band of western blot in figure 2.
The uncropped blot for figure 2B.
The uncropped blot for figure 2D.
