# Supplementary material for: The augment of regulatory T cells undermines the efficacy of anti-PD-L1 treatment in cervical cancer
Source: BMC Immunol. 2021 Sep 3;22:60. doi: 10.1186/s12865-021-00451-7 (PMC8414724; doi:10.1186/s12865-021-00451-7)
Supplement: Supplementary file 2 — Additional file 2. Treatment schedule and the representative figure for Cytometry flow analysis. [file 12865_2021_451_MOESM2_ESM.pptx]

## Slide 1
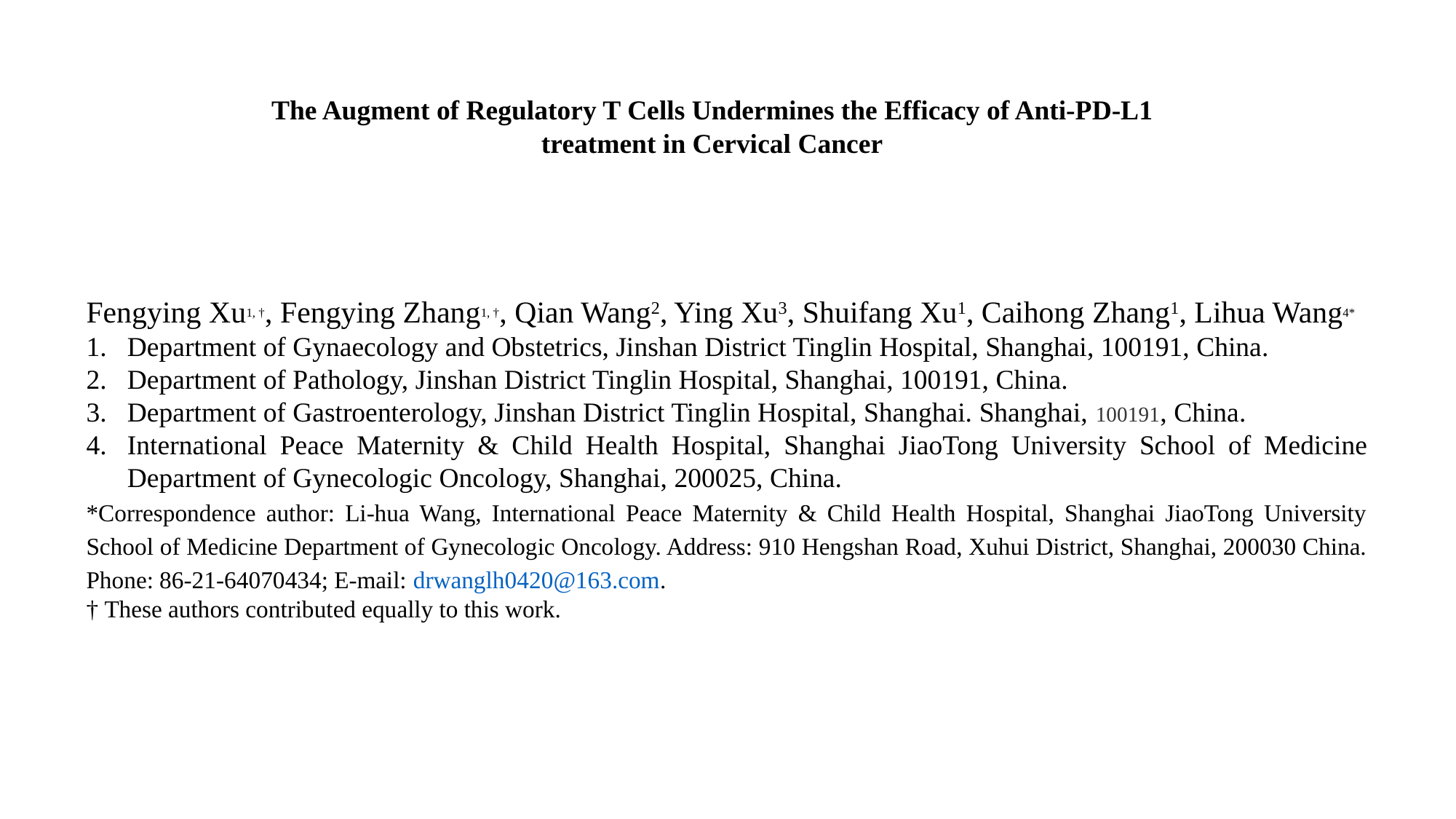

The Augment of Regulatory T Cells Undermines the Efficacy of Anti-PD-L1 treatment in Cervical Cancer
Fengying Xu1, †, Fengying Zhang1, †, Qian Wang2, Ying Xu3, Shuifang Xu1, Caihong Zhang1, Lihua Wang4*
Department of Gynaecology and Obstetrics, Jinshan District Tinglin Hospital, Shanghai, 100191, China.
Department of Pathology, Jinshan District Tinglin Hospital, Shanghai, 100191, China.
Department of Gastroenterology, Jinshan District Tinglin Hospital, Shanghai. Shanghai, 100191, China.
International Peace Maternity & Child Health Hospital, Shanghai JiaoTong University School of Medicine Department of Gynecologic Oncology, Shanghai, 200025, China.
*Correspondence author: Li-hua Wang, International Peace Maternity & Child Health Hospital, Shanghai JiaoTong University School of Medicine Department of Gynecologic Oncology. Address: 910 Hengshan Road, Xuhui District, Shanghai, 200030 China. Phone: 86-21-64070434; E-mail: drwanglh0420@163.com.
† These authors contributed equally to this work.

## Slide 2
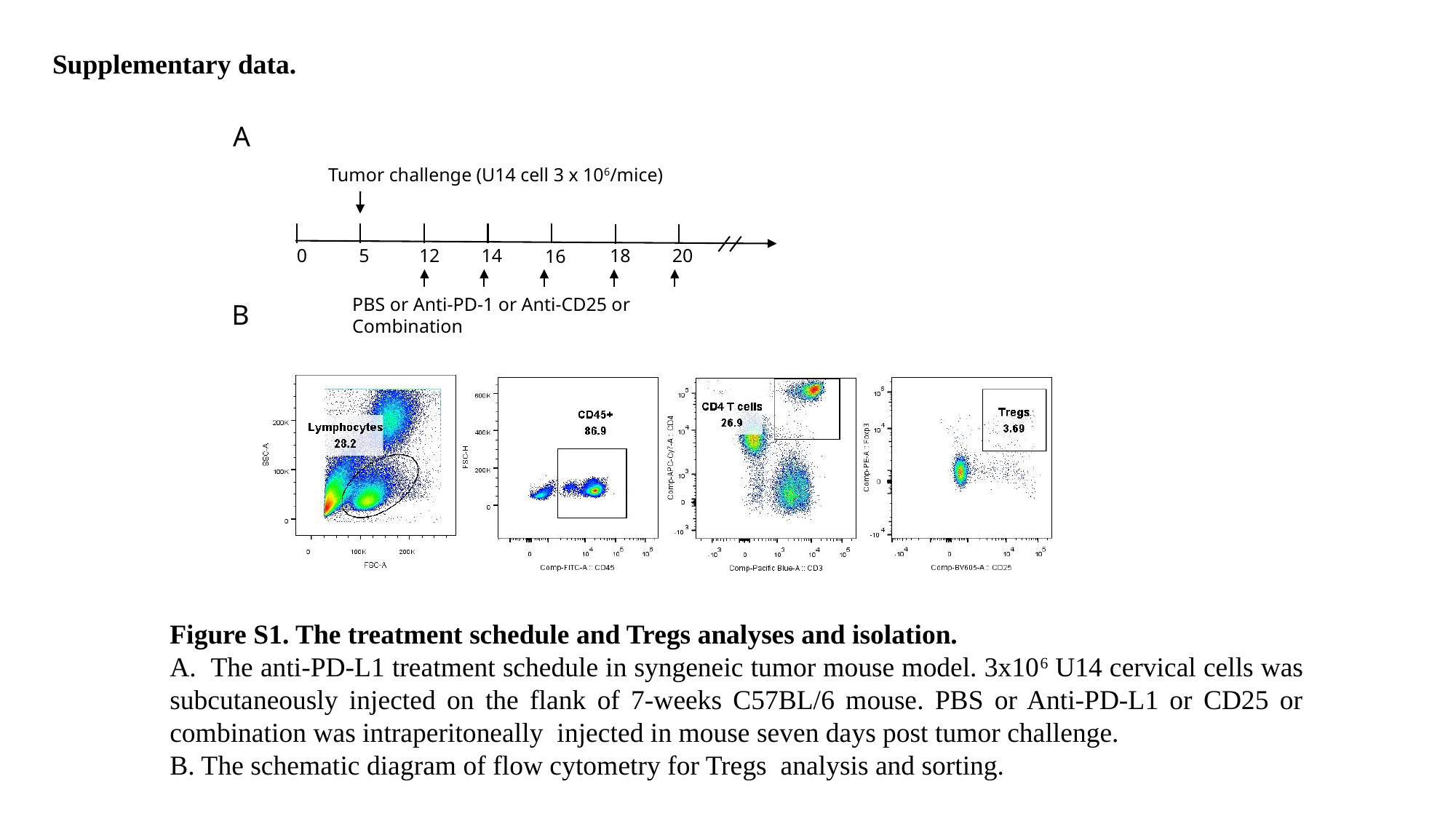

Supplementary data.
A
Tumor challenge (U14 cell 3 x 10^6^/mice)
0
5
12
14
18
20
16
PBS or Anti-PD-1 or Anti-CD25 or Combination
B
Figure S1. The treatment schedule and Tregs analyses and isolation.
The anti-PD-L1 treatment schedule in syngeneic tumor mouse model. 3x10^6^ U14 cervical cells was
subcutaneously injected on the flank of 7-weeks C57BL/6 mouse. PBS or Anti-PD-L1 or CD25 or combination was intraperitoneally injected in mouse seven days post tumor challenge.
B. The schematic diagram of flow cytometry for Tregs analysis and sorting.
A
